# TRPM8 contributes to liver regeneration via mitochondrial energy metabolism mediated by PGC1α

**DOI:** 10.1038/s41419-022-05475-4

**Published:** 2022-12-16

**Authors:** Xiaohua Lei, Qiang Liu, Wei Qin, Qing Tong, Zhenghao Li, Wendi Xu, Guoxing Liu, Jie Fu, Ju Zhang, Tao Kuang, Yaoli Shao, Chun Liu, Yu Fang, Zhenyu Cao, Likun Yan, Zhiqiang Liu, Siyuan Liu, Hirofumi Yamamoto, Masaki Mori, Xin M. Liang, Xundi Xu

**Affiliations:** 1 Hunan Provincial Key Laboratory of Hepatobiliary Disease Research & Division of Hepato-Biliary-Pancreatic Surgery, Department of Surgery, The Second Xiangya Hospital, Central South University, Changsha, Hunan People’s Republic of China; 2 The First Affiliated Hospital, Department of Hepato-Biliary-Pancreatic Surgery, Hengyang Medical School, University of South China, Hengyang, Hunan People’s Republic of China; 3 Department of Surgery, Gastroenterological Surgery, Graduate School of Medicine, Osaka University, Suita, Osaka Japan; 4 Department of Surgery and Science, Graduate School of Medical Sciences, Kyushu University, Fukuoka, Japan; 5 Wellman Center for Photomedicine, Division of Hematology and Oncology, Division of Endocrinology, Massachusetts General Hospital, VA Boston Healthcare System, Beth Israel Deaconess Medical Center, Harvard Medical School, Boston, MA USA; 6 Department of general surgery. Southern China Hospital, Health Science Center, Shenzhen University, Shenzhen, People’s Republic of China

**Keywords:** Cell growth, Genetics research

## Abstract

Impairment of liver regeneration leads to severe morbidity in acute and chronic severe liver disease. Transient receptor potential melastain 8 (TRPM8) is involved in a variety of processes, including temperature sensing, ion homeostasis, and cell proliferation. However, whether TRPM8 contributes to liver regeneration is still unclear. We assessed the effect and mechanism of TRPM8 in liver regeneration and hepatocyte proliferation in vivo and in vitro. In this study, we found that TRPM8 deficiency impairs liver regeneration in mice. Mechanistically, the results revealed that mitochondrial energy metabolism was attenuated in livers from TRPM8 knockout (KO) mice. Furthermore, we found that TRPM8 contributes to the proliferation of hepatocytes via PGC1α. Taken together, this study shows that TRPM8 contributes to liver regeneration in mice after hepatectomy. Genetic approaches and pharmacological approaches to regulate TRPM8 activity may be beneficial to the promotion of liver regeneration.

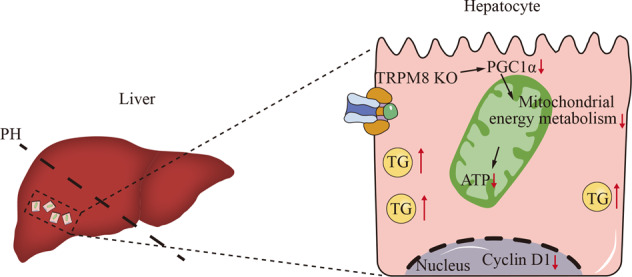

## Introduction

The liver has a great capacity for regeneration. In response to surgical resection, toxic exposure or viral injury, quiescent hepatocytes can be triggered to proliferate, which allows liver mass restoration and functional homeostasis [1–3]. Liver regeneration enables operations such as partial hepatectomy and liver transplantation. However, this ability to regenerate becomes unbearable in patients who underwent extensive partial liver resections, in patients with acute liver failure, or in patients who are in the setting of severe chronic liver injury with abnormal liver structure and significant liver fibrosis. No treatment strategy has been established to accelerate liver regeneration. Therefore, it is of great clinical significance to further understand the mechanism of liver regeneration.

Transient receptor potential melastain 8 (TRPM8), a member of the superfamily of TRP proteins, is involved in a variety of processes, including temperature sensing, calcium ion homeostasis, and cell proliferation [4, 5]. TRPM8 is a 128 kD protein that consists of 1104 amino acid residues. There is growing evidence that TRPM8 is associated with a variety of human diseases, suggesting that TRPM8 may play an important role in regulating cell proliferation [6]. TRPM8 mediated current and associated increase intracellular level of Ca^2+^ have been found in various types of cells. It is hypothesized that transient changes in intracellular level of Ca^2+^ lead to regulation of cell proliferation [6, 7]. However, whether TRPM8 contributes to liver regeneration is still unclear. Mitochondria are the major energy and metabolic centres at the cellular level. Most pathways involved in energy metabolism are completely or at least partially dependent on their function [8, 9]. Mitochondrial biogenesis maintains mitochondrial mass to restore energy homeostasis during energy deprivation. Mitochondrial functions, including reactive oxygen species regulation, calcium homeostasis and metabolism balance, are all involved in the liver regeneration process [10–14]. PGC1α, a master regulator of mitochondrial biogenesis and metabolic processes [15–17], is a transcriptional coactivator that acts as an inducible “booster” of nuclear receptors, enabling the body to meet energy requirements under different physiological and dietary conditions [18]. In liver, PGC1α mediates activation of PPAR target genes involved in hepatic fatty acid oxidation serving to increase ATP production. PPARα knockout mice showed impaired liver regeneration after hepatectomy, which is likely due to disordered hepatic lipid metabolism, cell cycle control and cytokine signalling [2, 19].

Based on the intriguing relationship between TRPM8 and cell proliferation revealed in previous studies, this report was undertaken to assess the influence of TRPM8 on 70% partial hepatectomy (PH), a well-known model for studying liver regeneration. We utilized in vivo and in vitro studies to demonstrate that TRPM8 deficiency impairs liver regeneration in mice after PH.

## Results

### Liver regeneration after partial hepatectomy is attenuated in TRPM8-deficient mice

**Fig. 1:**
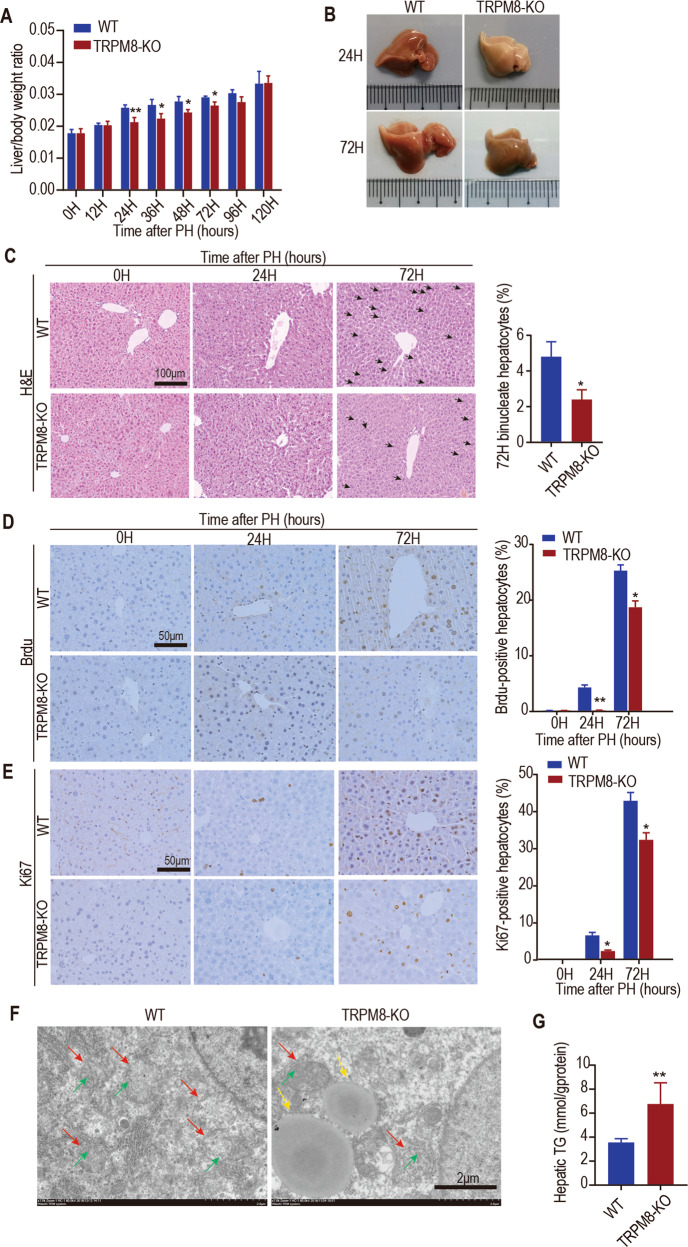
Liver regeneration after PH is attenuated in TRPM8-deficient mice. Male WT and TRPM8 KO mice at 10–12 weeks old were subjected to partial hepatectomy (PH). OH represents 0 h after PH. **A** The ratio of liver weight to body weight at the indicated time after PH is shown. **B** Morphological changes in the livers of WT mice and TRPM8 KO mice at 24 and 72 h after PH. **C** Representative H&E staining of liver Sections 0, 24, and 72 h after PH (left). Quantification of binucleated hepatocytes in mice 72 h after PH (right). The number of binucleated hepatocytes (the black arrowhead indicated) per 100 hepatocytes was calculated. **D**, **E** Immunohistochemical analysis of BrdU or Ki67 in paraffin tissues from livers of WT and TRPM8 KO mice at the indicated times after PH (left). Quantification of the percentage of BrdU- or Ki67-labelled nuclei (right). **F** Representative images of transmission electron microscopy at 24 h after the operation in mouse livers. Red arrow: mitochondria, yellow arrow: lipid drop, green arrow: mitochondrial cristae. **G** Elevation of plasma triglyceride (TG) levels in TRPM8 KO mice after PH. Data are presented as the mean ± standard error of the mean (SEM) (*n* = 5). *p < 0.05, **p < 0.01, vs. WT after PH at the indicated time.

To determine whether TRPM8 plays a role in liver regeneration, TRPM8 KO and wild-type (WT) littermates underwent partial hepatectomy, an in vivo model that has been widely used in liver regeneration studies [1]. Notably, compared with WT mice, TRPM8 KO mice showed a reduced ratio of liver to body weight after hepatectomy (Fig. 1A). The gross morphology showed that livers of WT mice became slightly pale at 24 h after PH, but livers of TRPM8 KO mice were obviously pale, indicating that hepatic steatosis may be more serious compared to WT mice. The livers of TRPM8 KO mice were smaller than the livers of WT mice at 72 h after PH (Fig. 1B). While examining the microscopic histology of livers, we found that TRPM8 KO mice had significantly fewer binucleate cells at 72 h postsurgery (Fig. 1C) and a significantly greater vacuolated area (Fig. 1C). In the next step, immunohistochemical analysis of Ki67 and BrdU, markers of cell proliferation, was performed. Our results revealed that the livers of TRPM8 KO mice possessed significantly fewer Ki67-positive cells and BrdU-positive cells than the livers of WT mice at hours 24 and 72 after PH (Fig. 1D, E). Scanning electron microscopy showed mitochondrial integrity loss and increased lipid accumulation in hepatocytes from TRPM8 KO mice at 24 h (Fig. 1F). Analysis of liver triglyceride (TG) content showed increased TG levels in the livers of TRPM8 KO mice compared with WT mice (Fig. 1G). Microscopic histology of livers also revealed that TRPM8 KO mice had significantly increased lipid accumulation in hepatocytes at 24 h after PH (Fig. S1A). As shown in (Fig. S1B), the lack of TRPM8 resulted in a significant elevation in serum ALT and AST levels after hepatectomy compared with WT mice, suggesting an impairment in liver function. TNF-α and IL-6 play important roles in the initiation of liver regeneration [20–22]. The TNF-α and IL-6 mRNA levels in the TRPM8 KO groups were significantly downregulated compared with the levels in the WT group at 24 h after PH (Fig. S1C). These data suggest that the loss of TRPM8 impairs the recovery of the liver after partial liver resection in mice.

### TRPM8 contributes to the proliferation of hepatocytes via mitochondrial energy metabolism

**Fig. 2:**
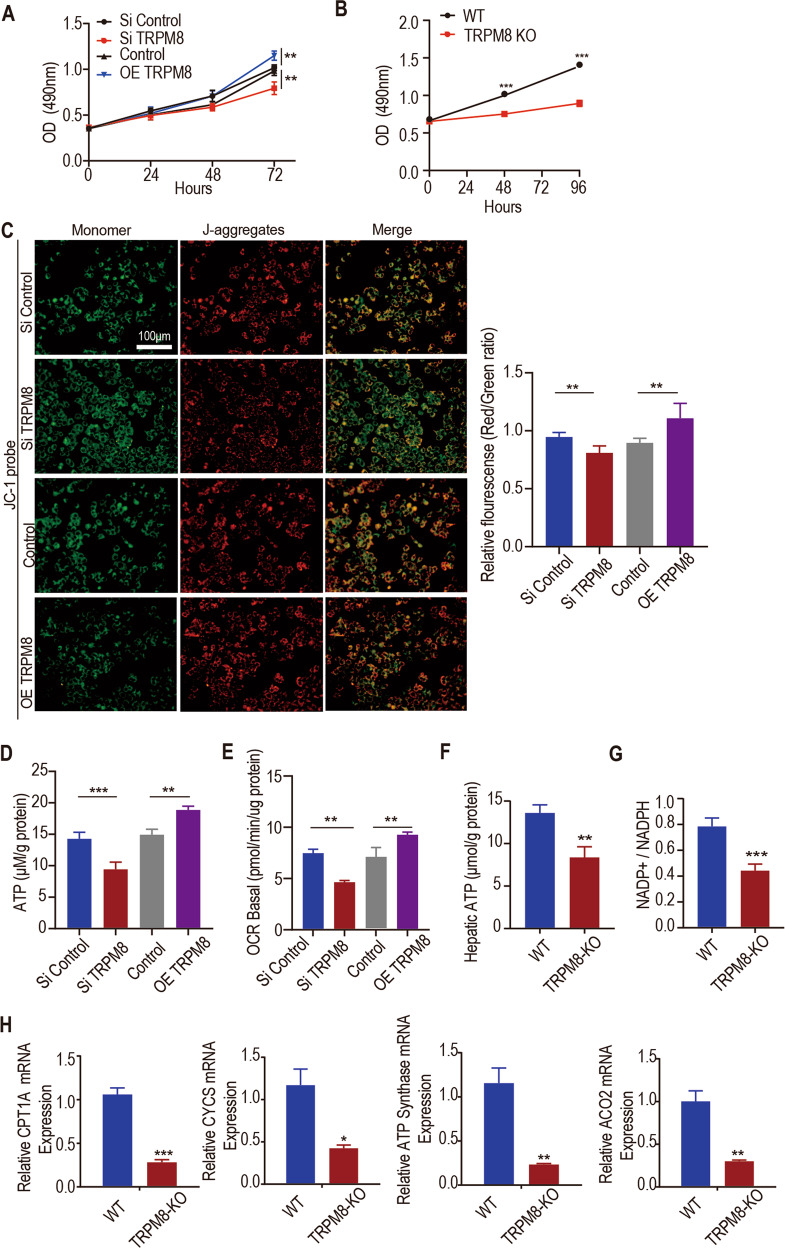
TRPM8 contributes to the proliferation of hepatocytes in vitro. **A** The effect of TRPM8-siRNA- or TRPM8-plasmid (overexpression, OE)-transfected L02 cells on proliferation was determined by MTT assays. **B** The proliferation of primary hepatocytes from the livers of WT and TRPM8 KO mice was determined by MTT assays (right). **C** The mitochondrial membrane potential of L02 cells was detected by JC-1 assays. **D** Biochemical detection of intracellular ATP levels in TRPM8-siRNA- or TRPM8-plasmid (OE)-transfected L02 cells. **E** OCR analysis of TRPM8-siRNA- or TRPM8-plasmid (OE)-transfected L02 cells. **F**, **G** Biochemical detection of liver intracellular ATP levels and NADP + /NADPH ratios and in TRPM8 KO and WT mice at 24 h after PH. **H** The mRNA levels of mitochondrial genes (CPT1Α, CYCS, ATP Synthase and ACO2) in the liver lysates of TRPM8 KO and WT mice 24 h after PH. The data are expressed as the mean ± SEM, *n* = 5. *p < 0.05, **p < 0.01, ***p < 0.001.

To investigate the effect of TRPM8 on the proliferation of the human hepatic cell Line L02, which is widely used in the study of hepatocyte proliferation and liver regeneration [23–25], we manipulated TRPM8 expression in L02 cells by small interfering RNA (siRNA) knockdown and overexpression. Three siRNAs (siTRPM8-1, siTRPM8-2, and siTRPM8-3) were designed to silence TRPM8 expression and were subsequently named siTRPM8. The expression level of TRPM8 was identified by real-time polymerase chain reaction (PCR) and Western blotting (Fig.S2A-B). siTRPM8-1 was the most effective siRNA and was chosen for further study. Compared to the Si Control, Si TRPM8 had a lower absorbance in the methyl thiazol tetrazolium (MTT) assay, which indicated a lower proliferation rate (Fig. 2A). In contrast, overexpression of TRPM8 in L02 cells resulted in higher absorbance (Fig. 2A). The MTT results were verified in primary hepatocytes (Fig. 2B). These findings were consistent with the results obtained by the EdU staining assay (Fig. S2C), suggesting that TRPM8 is involved in hepatocyte proliferation. Cell cycle analysis showed that knockdown of TRPM8 in L02 cells significantly suppressed the number of cells in the S phase. Overexpression of TRPM8 in L02 cells significantly increased the number of cells in the S phase (Fig. S2D). The effect of TRPM8 in hepatocytes was further investigated by examining mitochondrial depolarization. Si TRPM8 L02 cells stained with JC-1 emitted fluorescent colour changes from orange-red to green, which indicated a decrease in mitochondrial membrane potential, which is a landmark event in the early stage of apoptosis (Fig. 2C). However, overexpression of TRPM8 in L02 cells had the opposite effect (Fig. 2C). ATP production was enhanced upon TRPM8 overexpression but significantly attenuated upon TRPM8 knockdown (Fig. 2D). Mitochondrial ROS levels were measured by Mito-SOX staining on TRPM8 gene-modified hepatocytes. The results showed that TRPM8 knockdown increased the mitochondrial ROS levels and that TRPM8 overexpression had the opposite effect (Fig. S2E). Attenuated mitochondrial activity could be induced by downregulation of TRPM8. To confirm this downregulation, mitochondrial activity and metabolism were measured by using a Seahorse analyser to detect the O_2_ consumption rates of TRPM8 gene-modified hepatocytes. The results showed that TRPM8 knockdown decreased the basal O_2_ consumption rate and that TRPM8 overexpression had the opposite effect (Fig. 2E).

Cell proliferation processes, including DNA replication, require energy. To explore the energy production in the liver tissues after PH, we measured the NADP+/NADPH ratio and ATP content of the liver tissues 24 h after surgery. We found that the ATP content and NADP+/NADPH ratio of TRPM8 KO mice were significantly lower than the ratios of WT mice (Fig. 2F, G). Quantitative PCR (qPCR) assays showed that mitochondrial genes (CPT1Α, CYCS, ATP Synthase and ACO2) involved in oxidative phosphorylation and the TCA cycle were downregulated in the livers of TRPM8 KO mice compared with WT mice (Fig. 2H).

### TRPM8 plays a role in liver regeneration and hepatocyte proliferation through mitochondrial biogenesis mediated by PGC1α

**Fig. 3:**
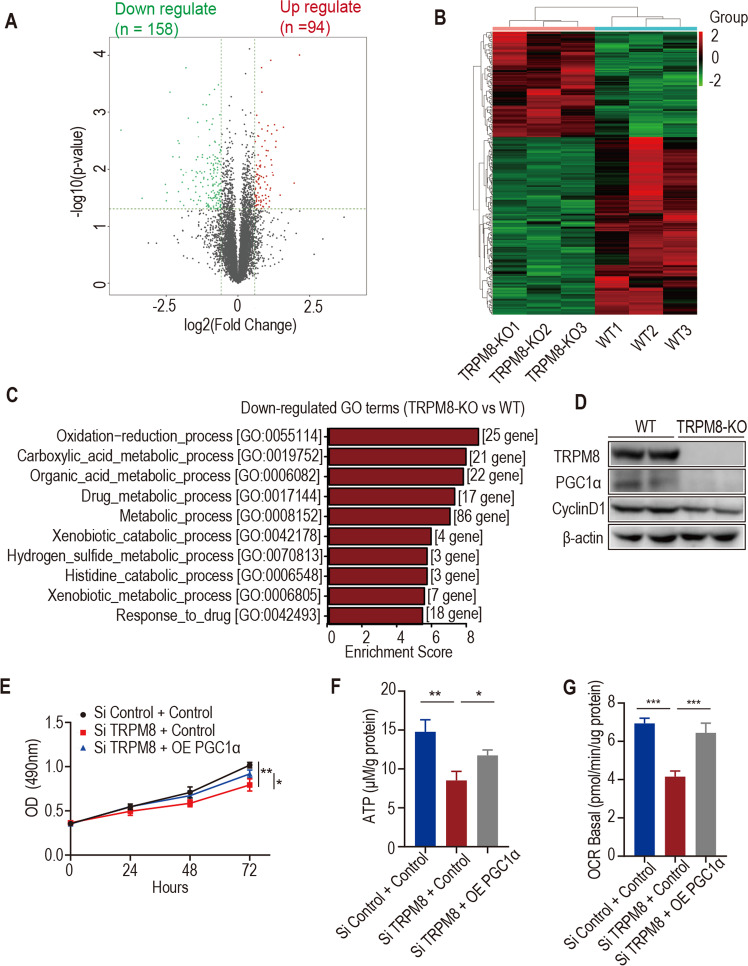
mRNA-seq and bioinformatics analysis revealed that metabolic processes were attenuated in TRPM8 KO mouse livers. **A**, **B** Volcano plot and heatmap presentation of significantly downregulated and upregulated genes in the livers of TRPM8 KO mice compared to WT mice 24 h after PH. **C** GO enrichment analysis of the downregulated genes in the livers of TRPM8 KO mice compared to WT mice 24 h after PH. **D** The protein levels of TRPM8, PGC1α and CyclinD1 in the livers of WT and TRPM8 KO mice. **E** The proliferation of TRPM8-siRNA- and PGC1α-plasmid (OE) cotransfected L02 cells was determined by MTT assays. **F** The intracellular ATP levels in TRPM8-siRNA- and PGC1α-plasmid (OE)-cotransfected L02 cells. **G** OCR analysis of TRPM8-siRNA- and PGC1α-plasmid (OE)-cotransfected L02 cells. **H** The protein levels of TRPM8 and PGC1α in L02 cell lysates after TRPM8 knockdown or TRPM8 overexpression. Data are presented as the mean ± SEM (*n* = 5). *p < 0.05, **p < 0.01, ***p < 0.01.

To determine the mechanism of TRPM8 in liver regeneration, liver tissues were collected from TRPM8 KO and WT mice at 24 h after PH, and mRNA-seq was carried out. We observed that the ratio of liver to body weight alteration between these two groups began at 24 h after PH and was most dramatic, therefore, we would like to use this time point for mRNA-seq. We showed that 94 genes were upregulated and 158 genes were downregulated in TRPM8 KO mice (Fig. 3A, B). To account for the transcriptome changes regulated by TRPM8, we used two database platforms (Kyoto Encyclopedia of Genes and Genomes (KEGG) and Gene Ontology (GO)) for pathway enrichment analysis. TRPM8-related pathway genes were involved in oxidation reduction and metabolic processes (Fig. 3C). PGC1α, one of downregulated genes in TRPM8 KO mice, is an overlapping gene of GO entries, including oxidation-reduction process, metabolic process and cell proliferation process. PGC1α is a master regulator of mitochondrial biogenesis and metabolic processes [15–17]. Many studies have shown that PGC1a plays a key role in inducing mitochondrial gene expression of oxidative phosphorylation and the tricarboxylic acid (TCA) cycle in various tissues [26–28], confirming the reduced expression of PGC1α in liver of TRPM8 KO mice compared to WT mice (Fig. 3D). Western blotting analysis revealed that the level of PGC1α decreased in the lysates of TRPM8 knockdown cells while increased in the lysates of TRPM8 overexpression L02 cells (Fig. S3A). Next, we detected the effect of TRPM8 on the intracellular Ca^2+^ concentration. The fluorescence intensity was significantly enhanced upon TRPM8 overexpression but attenuated upon TRPM8 knockdown in L02 cells (Fig. S3B). The increase in intracellular Ca^2+^ may activate expression of PGC1α [29]. Mitochondrial biogenesis related proteins (NRF1, TFAM, ACO2 and ATP5A) were downregulated in liver of TRPM8 KO mice compared to WT mice (Fig. S3C). The copy number of mitochondrial DNA was decreased in liver of TRPM8 KO mice compared to WT mice (Fig. S3D).

To further test whether TRPM8 contributes to hepatocyte proliferation via PGC1α, the plasmid PGC1α was co-transfected into TRPM8 knockdown L02 cells. Overexpression and silencing efficacy were identified by real-time PCR and Western blotting. The MTT and ATP production assays indicated that overexpression of PGC1α rescue the effect of TRPM8 knockdown to promote cell proliferation and ATP production (Fig. 3E, F). Mitochondrial activity assays revealed that overexpression of PGC1α rescued the effect of TRPM8 knockdown on promoting the basal O_2_ consumption rate (Fig. 3G).

### TRPM8 antagonist (M8-B) impairs liver regeneration through mitochondrial energy metabolism after partial hepatectomy in mice

**Fig. 4:**
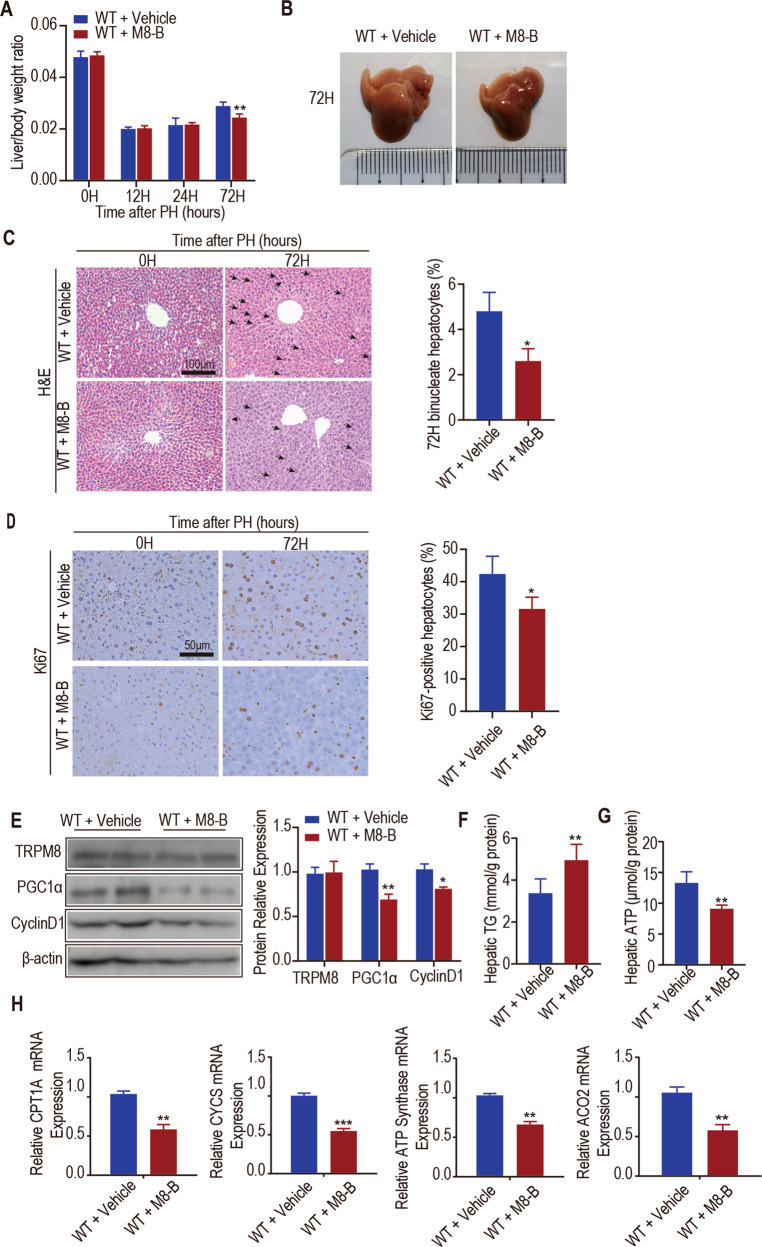
Pharmacological effects of a TRPM8 antagonist (M8-B) on liver regeneration. Male WT mice at 10–12 weeks old were subjected to partial hepatectomy (PH) and treated with vehicle or TRPM8 antagonist (M8-B. **A** The ratio of liver weight to body weight at the indicated time after PH is shown. **B** Morphological changes in the livers of mice at 72 h after PH. **C** Representative H&E staining of liver sections 0 and 72 h after PH (left). Quantification of binucleated hepatocytes in mice 72 h after PH (right). The number of binucleated hepatocytes (the black arrowhead indicated) per 100 hepatocytes was calculated. **D** Immunohistochemical analysis of Ki67 in paraffin tissues from livers at the indicated times after PH (Left). Quantification of the percentage of Ki67-labelled nuclei (right). **E** The protein levels of TRPM8, PGC1α and CyclinD1 in the liver lysates of mice 24 h after PH (Left). Quantification of the proteins expression (right). **F**, **G** Biochemical detection of liver TG content and intracellular ATP levels at 24 h after PH. **H** The mRNA levels of mitochondrial genes (CPT1Α, CYCS, ATP Synthase and ACO_2_) in the liver lysates of mice 24 h after PH. Data are presented as the mean ± SEM (*n* = 5). *p < 0.05, **p < 0.01, ***p < 0.01 vs. WT after PH at the indicated time.

Given that M8-B is a selective and potent antagonist of the TRPM8 channel in vivo and in vitro [30, 31], we hypothesized that M8-B may impair liver regeneration after PH. Therefore, the WT mice were treated with M8-B (6.0 mg/kg, ip, daily) after PH. Compared with mice that received vehicle, mice treated with M8-B showed a reduced ratio of liver to body weight after hepatectomy (Fig. 4A), a significant elevation in serum ALT and AST levels (Fig. S4A), a smaller liver (Fig. 4B), significantly fewer binucleate cells (Fig. 4C) and Ki67-positive cells at 72 h post-operation (Fig. 4D). Western blotting analysis revealed that the levels of PGC1α and CyclinD1 were decreased in the liver lysates of mice treated with M8-B compared with mice that received vehicle 24 h after PH (Fig. 4E).

Analysis of liver TG content showed increased TG levels in the livers of mice treated with M8-B compared with vehicle-treated mice (Fig. 4F). We found that the hepatic ATP content from M8-B-treated mice was significantly lower than the hepatic ATP content of vehicle-treated mice 24 h after PH (Fig. 4G). qPCR assays showed that mitochondrial gene targets of PGC1a involved in oxidative phosphorylation and the TCA cycle were downregulated in the livers of mice treated with M8-B compared with vehicle-treated mice at 24 h after PH (Fig. 4H).

The TNF-α and IL-6 mRNA levels in the livers of mice treated with M8-B were significantly downregulated compared with the levels in the vehicle-treated mice at 24 h after PH (Fig. S4B).

### Activation of PGC1α partially restores liver regeneration after partial hepatectomy in TRPM8 KO mice

**Fig. 5:**
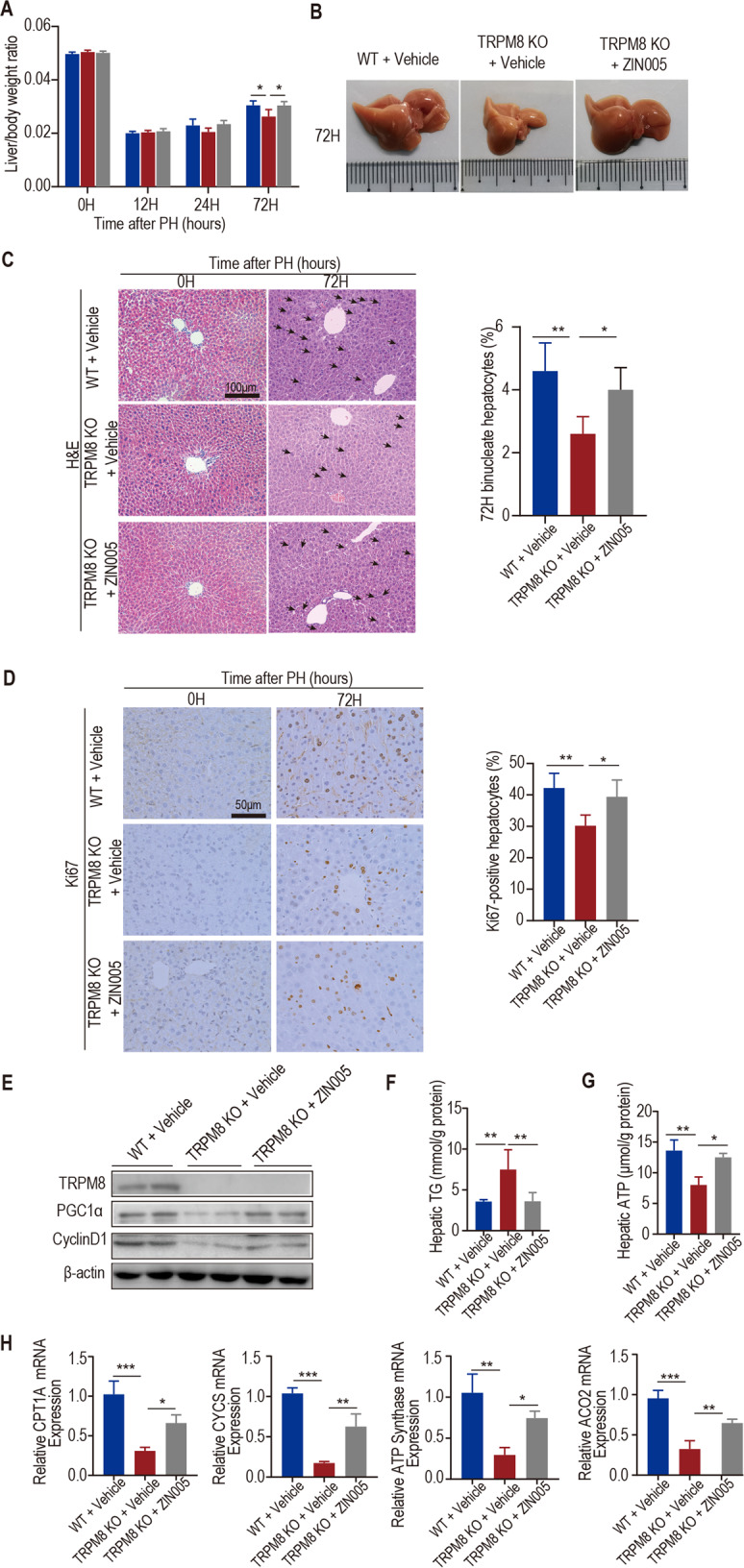
Pharmacological effects of a PGC1α agonist (Zln005) on liver regeneration. Male WT and TRPM8 KO mice at 10–12 weeks old were subjected to partial hepatectomy (PH). WT mice were treated with vehicle, and TRPM8 KO mice were treated with vehicle or a PGC1α agonist (Zln005). **A** The ratio of liver weight to body weight at the indicated time after PH is shown. **B** Morphological changes in the livers of mice at 72 h after PH. **C** Representative H&E staining of liver sections 0 and 72 h after PH (left). Quantification of binucleated hepatocytes in mice 72 h after PH (right). The number of binucleated hepatocytes (the black arrowhead indicated) per 100 hepatocytes was calculated. **D** Immunohistochemical analysis of Ki67 in paraffin tissues from livers at the indicated times after PH (Left). Quantification of the percentage of Ki67-labelled nuclei (right). **E** The protein levels of TRPM8, PGC1α and CyclinD1 in the liver lysates of mice 24 h after PH. **F**, **G** Biochemical detection of liver TG content and intracellular ATP levels at 24 h after PH. **H** The mRNA levels of mitochondrial genes (CPT1Α, CYCS, ATP Synthase and ACO_2_) in the liver lysates of mice 24 h after PH. Data are presented as the mean ± SEM (*n* = 5). *p < 0.05, **p < 0.01, ***p < 0.01 vs. WT after PH at the indicated time.

To further study whether TRPM8 contributes to liver regeneration via PGC1α in vivo, Zln005, as a selective PGC1α agonist, was administered to TRPM8 KO mice after PH daily. We found that compared with TRPM8 KO mice that received vehicle, TRPM8 KO mice treated with Zln005 showed a higher ratio of liver to body weight after hepatectomy (Fig. 5A), significantly decreased ALT and AST levels in plasma (Fig. S5A), larger liver (Fig. 5B), and obviously more binucleate cells (Fig. 5C) and Ki67-positive cells at 72 h post-operation (Fig. 5D). Western blotting analysis revealed that the levels of PGC1α and CyclinD1 were increased in the liver lysates of mice treated with Zln005 compared with mice that received vehicle 24 h after PH (Fig. 5E). Analysis of liver TG content showed decreased TG levels in the livers of TRPM8 KO mice treated with Zln005 compared with TRPM8 KO mice that received vehicle 24 h after PH (Fig. 5F). We found that the hepatic ATP content from TRPM8 KO mice treated with Zln005 was significantly more than the hepatic ATP content of TRPM8 KO mice that received vehicle 24 h after PH (Fig. 5G). qPCR assays showed that mitochondrial gene targets of PGC1α were upregulated in the livers of TRPM8 KO mice treated with Zln005 compared with TRPM8 KO mice that received vehicle at 24 h after PH (Fig. 5H). TNF-α and IL-6 mRNA levels in the livers of TRPM8 KO mice treated with Zln005 were significantly upregulated compared with the levels in TRPM8 KO mice that received vehicle at 24 h after PH (Fig. S5B).

These data strongly suggest that the PGC1α agonist partially restores the effect of TRPM8 deficiency in mice, indicating that TRPM8 contributes to liver regeneration by regulating the expression of PGC1α.

## Discussion

The liver possesses an extraordinary ability to regenerate. Hepatocellular organelle and cytokines respond in hours, and the liver is capable of restoring its lost mass in days after hepatectomy [32]. Mitochondria are bioenergetic, biosynthetic and signalling organelles that are integral in stress sensing to allow for cellular adaptation to the environment [33]. In liver regeneration, mitochondrial regulation of oxygen uptake, reactive oxygen species content, intracellular calcium homeostasis and energy metabolism are all involved in the proliferation and apoptosis of multiple liver cells [10–14]. The regenerative process is a complex phenomenon characterized by the proliferation of fully differentiated hepatic cells, during which the liver performs all its normal functions. The clinical relevance of TRPM8 in in liver regeneration has not previously been investigated. Our work demonstrated that TRPM8 ablation disrupted the structure of hepatocellular mitochondria together with its dysfunctions, including ATP production, oxygen consumption and membrane potential, highlighting its contributions to liver regeneration.

In vitro and in vivo assays demonstrated that TRPM8 exerts an effect on liver regeneration through a PGC1α signalling interaction. Peroxisome proliferator-activated receptor γ (PPAR-γ) coactivator 1α (PGC1α) is an important coactivator of several nuclear receptors regulating mitochondrial function in various tissues, including the liver, heart, skeletal muscle, and brain. PGC1α, initially discovered as one of the PPAR-γ-binding proteins present in brown adipose tissue as a response to cold treatment, is positively related to the fatty acid oxidation reduction process, metabolic process and cell proliferation process as well as the inflammatory response [17, 18, 34]. In agreement with a previous report [35], TRPM8 inhibition ameliorated mitochondrial biogenesis represented by PGC1α. There are several lines of evidence supporting the identification of TRPM8 contributes to liver regeneration via PGC1α. First, the results showed that the level of PGC1α protein was decreased in the liver lysates of TRPM8 KO mice compared with WT mice after PH. Second, TRPM8 gain and loss of function approaches in vitro studies including JC-1 probe assay, basal O_2_ consumption rate and ATP production test demonstrated TRPM8 was associated with mitochondrial biogenesis, in which PGC1α is a master regulator. Third, the rescue assay showed that overexpression of PGC1α could rescue the inhibition effect of silencing of TRPM8 on liver cells. Furthermore, in vivo study PGC1α agonist was found to partially restore the effect of the deficiency of TRPM8 in liver regeneration. Those findings suggest that TRPM8 plays important roles in acute-phase liver regeneration, which appears to be critical for PGC1α protein with mitochondrial O_2_ consumption and ATP production biogenesis. TRPM8 is an energy metabolism signal critical for liver growth and liver regeneration after injury.

### Summary

In summary, this study identifies that TRPM8 contributes to liver regeneration in mice after hepatectomy. Genetic approaches and pharmacological approaches to regulate TRPM8 activity may be beneficial to the promotion of liver regeneration in patients who underwent extensive partial liver resections or in patients with severely damaged livers.

## Materials and methods

### Animals and partial hepatectomy

TRPM8 KO mice and wild-type (WT) C57BL/6 mice were purchased from the Jackson Laboratory (ME, USA). All mice were maintained in a specific pathogen-free facility and fed ad libitum on a standard pellet diet and pure water at the animal experimental centre of the Second Xiangya Hospital of Central South University. They were kept under constant temperature and humidity in a 12-h controlled dark/light cycle. Male mice at the age of 10–12 weeks were subjected to 70% partial hepatectomy (PH) under sodium pentobarbital anaesthesia as described [36]. The left lateral and median hepatic lobes were ligated before resection. After the abdominal cavity was closed, the sutured incision was sterilized with betaine. Liver samples were collected, either fixed in buffered formalin or snap frozen in liquid nitrogen, and stored at −80 °C until use after sacrifice. Postoperative weight and liver weight were measured. All animal experiments were performed in accordance with the National Institutes of Health Guidelines for the Care and Use of Laboratory Animals and were approved by the Central South University Institutional Animal Care and Use Committee (Changsha, China).

### Cell line, primary hepatocyte and cell culture

The immortalized human hepatic cell Line L02 was purchased from the Cell Bank of Typical Culture Preservation Committee of the Chinese Academy of Science, Shanghai, China. The cell line was authenticated and confirmed negative for mycoplasma contamination by the provider. Primary hepatocytes were obtained by rapid separation from the livers of TRPM8 KO and WT mice as described [37]. The cells were cultured in Dulbecco’s modified eagle’s medium containing 1 mg/mL D-glucose and supplemented with 0.3 mg/mL L-glutamine and 10% foetal bovine serum (FBS) (Gibco, CA, USA). All cells were maintained in a 5% CO_2_ humidified incubator at 37 °C.

### Transmission electron microscopy

Liver tissues obtained at 24 h after operation from TRPM8 KO and WT mice were sectioned into 0.1–0.2 mm^3^ small chips and fixed in 2.5% glutaraldehyde. Transmission electron microscopy (TEM) was used for examination, as previously described [36]. Three mice were used in each group.

Further details of the materials and methods are described in the Supplementary Materials and Methods.

### Data sharing

For original data, please email the corresponding author Xundi Xu.

## Supplementary information

Supplementary Materials and Methods

Supplementary Figure S1

Supplementary Figure S2

Supplementary Figure S3

Supplementary Figure S4

Supplementary Figure S5

Original Data File

checklist
