# Correction: TRPM8 contributes to liver regeneration via mitochondrial energy metabolism mediated by PGC1α

**DOI:** 10.1038/s41419-023-05703-5

**Published:** 2023-03-20

**Authors:** Xiaohua Lei, Qiang Liu, Wei Qin, Qing Tong, Zhenghao Li, Wendi Xu, Guoxing Liu, Jie Fu, Ju Zhang, Tao Kuang, Yaoli Shao, Chun Liu, Yu Fang, Zhenyu Cao, Likun Yan, Zhiqiang Liu, Siyuan Liu, Hirofumi Yamamoto, Masaki Mori, Xin M. Liang, Xundi Xu

**Affiliations:** 1 Hunan Provincial Key Laboratory of Hepatobiliary Disease Research & Division of Hepato-Biliary-Pancreatic Surgery, Department of Surgery, The Second Xiangya Hospital, Central South University, Changsha, Hunan People’s Republic of China; 2 The First Affiliated Hospital, Department of Hepato-Biliary-Pancreatic Surgery, Hengyang Medical School, University of South China, Hengyang, Hunan People’s Republic of China; 3 Department of Surgery, Gastroenterological Surgery, Graduate School of Medicine, Osaka University, Suita, Osaka Japan; 4 Department of Surgery and Science, Graduate School of Medical Sciences, Kyushu University, Fukuoka, Japan; 5 Wellman Center for Photomedicine, Division of Hematology and Oncology, Division of Endocrinology, Massachusetts General Hospital, VA Boston Healthcare System, Beth Israel Deaconess Medical Center, Harvard Medical School, Boston, MA USA; 6 Department of general surgery. Southern China Hospital, Health Science Center, Shenzhen University, Shenzhen, People’s Republic of China

**Keywords:** Cell growth, Genetics research

Correction to: *Cell Death and Disease* 10.1038/s41419-022-05475-4 published online 16 December 2022

The original version of this article contained an error in an author name. The correct form should be Xin M. Liang not Xinmin Liang. The original article has been corrected.

