# Supplementary material for: TRPM8 contributes to liver regeneration via mitochondrial energy metabolism mediated by PGC1α
Source: Cell Death Dis. 2022 Dec 16;13(12):1050. doi: 10.1038/s41419-022-05475-4 (PMC9758188; doi:10.1038/s41419-022-05475-4)
Supplement: Supplementary file 1 — Supplementary Materials and Methods [file 41419_2022_5475_MOESM1_ESM.docx]

**Supplementary Materials and Methods**

**Mice blood sample collection and biochemical measurements**

Blood samples were collected from each mouse at several specified time points after the surgery. Samples with the addition of heparin were used to assess platelet count. Samples without heparin were centrifuged at 10000 RPM for 10 min to collect the serum. 50 μL serum was used to measure serum concentrations of alanine aminotransferase (ALT), aspartic aminotransferase (AST) and Triglyceride (TG) with IDEXX Vet Test Chemistry Analyzer (IDEXX Laboratories Inc. ME, USA). The residual serum was stored at -80℃ for subsequent experiments.

**Immunohistochemistry**

Immunohistochemical staining for tissue was performed using the polymer HRP detection system (Zhongshan Goldenbridge Biotechnology, Beijing, China) on formalin-fixed, paraffin-embedded tissue sections that had been cut to 4-µm thickness. Briefly, the liver sections were incubated with diluted primary antibody against Brdu (1:200 dilution) or Ki67 (1:200 dilution) overnight in a humidification chamber at 4℃. and then HRP conjugated secondary antibody secondary antibody at room temperature for 30min. The solution of diaminobenzidine was used as the color reagent to redye. Immunostaining negative control was prepared by omitting primary antibody. The positive cells were scored in at least 5 fields at 400× magnification, and the data were expressed as mean ±SD. There were five mice in each group. For assessment of 5-bromo-2-deoxyuridine incorporation, mice were intraperitoneally injected with 100 mg/kg 5-bromo-2-deoxyuridine (BrdU) purchased from Sigma-Aldrich (MA, USA) two hours before euthanasia.

**mRNA-seq**

RNA-seq and bioinformatics analysis was carried at Aksomics Co., Ltd. Briefly, total RNA isolated with TRIzol reagent was treated with RNase-free DNaseI (New England BioLabs, MA, USA) at 37℃ for 10 min. The NEBNext® Poly(A) mRNA Magnetic Isolation Module (New England Biolabs, MA, USA) was used to isolate mRNA from the total RNA samples. RNA-seq libraries were sequenced as paired-end 100 bp sequence tags using the standard Illumina HiSeq 4000 pipeline (https://doi.org/10.6084/m9.figshare.20384367).

**Western blotting**

Total proteins were extracted with RIPA lysis buffer and separated by 10% sodium dodecyl sulfate SDS-PAGE and then transferred to the polyvinylidene difluoride (PVDF) membrane purchased from Millipore Corporation (MA, USA). The membrane was blocked with 5% skimmed milk and incubated with the primary antibody, followed by incubation with a secondary antibody. The blots were visualized by enhanced chemiluminescence (ThermoFisher Scientific, MA, USA). The antibodies used are listed as follows: Antibody against TRPM8 (GTX54866) was purchased from GeneTex (California, USA). Antibody against PGC1α (AB3242) was purchased from Sigma-Aldrich (St. Louis, USA). Antibody against TFAM (AF0531) was purchased from Affinity Biosciences (Cincinnati, USA). Antibodies against CylinD1 (60186-1-Ig), NRF1 (12482-1-AP), ACO2 (11134-1-AP), ATP5A1 (14676-1-AP) and β-actin (60008-1-AP) were purchased from Proteintech (Rosemont, USA).

**Quantitative real-time polymerase chain reaction (qRT-PCR)**

A piece of liver tissue about 25 mg was homogenized in 1000 μL TRIzol®Reagent (Thermo Scientific, MA, USA). Total RNA was extracted according to the manufacturer's instructions. To measure mRNA expression, qRT-PCR was performed using the LightCycler®96 system (Roche, Basel, Switzerland). The target genes amplification were normalized with β-actin amplification level as the endogenous control. Data analysis and calculation was carried out using 2-ΔΔCT comparison method described in the manufacturer. The primers are shown in Table.

| Gene Primer sequence |
| --- |
| TNFα F: AGCACAGAAAGCATGATCCG  R: CACCCCGAAGTTCAGTAGACA  IL6 F: GACTTCCATCCAGTTGCCTT  R: ATGTGTAATTAAGCCTCCGACT  Cycs F: GAACAAGTGTGGTTGCACCG  R: TAATTCGTTCCGGGCTGGTC  CPT1α F: CTTCAATACTTCCCGCATCCCT  R: AGCAGCCTCCCGTCATGGTA  ATP Synthase F: ACAACATCTTCCGCTTTACCCAG  R: CACATAGATAGCCTGCACCGAG  aco2 F: GTTGAACCGGCCTCTTACTCT  R: CAACCTGGGCCTCAATCAGA |

**Cell transfection**

TRPM8, PGC1α overexpression of plasmid and its negative control were purchased from GeneChem (Shanghai, China). TRPM8, PGC1α knockdown of small interfering RNA (SiRNA) and its negative control were purchased from RIBO (Guangzhou, China). overexpression plasmid or SiRNA was transfected into L02 cells using Lipofectamine™ 3000 transfection reagent (Invitrogen, MA, USA) according to the instructions. To test whether PGC1α was regulated by TRPM8, PGC1α overexpression of plasmid (GeneChem, shanghai, china) was co-transfected TRPM8 siRNA into cells. After 6 hours of transfection, the medium was changed to a fresh medium containing 10% FBS for further incubation. After 48 hours of transfection, cells were collected for further study. Transfection efficacy were identified by real-time PCR and western blotting.

**MTT assay**

After transfection, cell viability was measured by MTT assay. L02 cells (5 × 10^3^) were seeded into in 96-well plates and stained at the indicated time points with 20 μL sterile MTT (5 mg/mL) (Sigma-Aldrich, MA, USA) at 37 °C for 4 h, followed by removal of the culture medium and the addition of 150 μL DMSO (Sigma-Aldrich, MA, USA), followed by the measurement of the absorbance at 490 nm. All studies were conducted with three replicates.

**EDU assay**

After transfection, L02 cells were seeded into 96-well plates. The cell proliferation rate was detected by a 5-ethynyl-20-deoxyuridine (EdU) kit (RiboBio, Guangzhou, China), according to the manufacturer’s instructions. 100 μL culture medium containing 50μM EdU was added to each well, and incubated for 2 h. In addition, 30 min after fixation with 4% paraformaldehyde, followed by a 10-min treatment with 0.5% Triton, and then 1 x Apollo reaction cocktail (RiboBio,Guangzhou,China) was added to the cells and incubated for 30 min. Furthermore, the cells were stained with Hoechst 33342 for 30 min for DNA content analysis. Finally, the EdU-stained cells were visualized under a fluorescence microscope (Olympus, Tokyo, Japan). Assays were performed three times using triplicate wells.

**Flow cytometry analysis**

For cell cycle analysis, cells were washed two times in ice-cold PBS, resuspended in 1 ml ice-cold 70% ethanol with gentle vortex and incubated overnight at 4 °C. Cells were then washed with 1X PBS and resuspended in buffer containing 10 μg/ml propidium iodide, 10μg/ml RNase A and 0.1% Triton X-100 and incubated for 30 min at room temperature in the dark. After incubation, the DNA content in the cells were analyzed by flow cytometry

**Mitochondrial membrane potential detection**

JC-1 probe (Beyotime, Jiangsu, China) was employed to measure mitochondrial depolarization in L02 cells. Briefly, Cells cultured in six-well plates after indicated treatments were incubated with an equal volume of JC-1 staining solution (5 μg/ml) at 37 °C for 20 min and washed twice with JC-1 staining buffer twice. Mitochondrial membrane potentials were monitored by determining the relative amounts of dual emissions from mitochondrial JC-1 monomers or aggregates using a fluorescent microscope (Olympus, Tokyo, Japan).

**Measurement of intracellular Ca^2+^**

Fluo-4 AM Ca^2+^ probe (Beyotime, Jiangsu, China) was employed for measuring the intracellular Ca^2+^ concentration in L02 cells. Briefly, Cells were cultured in six-well plates after indicated treatments and incubated with Fluo-4 AM according to manufacturer’s instruction. Cells were then imaged by inverted fluorescence microscope (Olympus, Tokyo, Japan).

**Measurement of mitochondrial ROS**

Mito-sox staining was used to detect mitochondrial ROS of TRPM8 gene-modified hepatocytes. Simply put, L02 cells were grown on 24-well plates with glass cladding after different treatments. Then, the L02 cells were incubated with 5 μM Mito-SOX (Invitrogen, MA, USA) in darkness at 37℃ for 15 min. Finally, the cells were photographed under microscope and the fluorescence intensity was analyzed from five different view fields of each group with ImageJ software.

**Detection lipids with oil Red O staining**

Oil Red O staining test kit (C0158S, Beyotime, Jiangsu, China) was used to detect lipids in L02 cells according to the manufacturer’s instructions. Briefly, L02 cells were seeded into 12-well plates after transfection. Fixated with 4% paraformaldehyde for 10min after the cells grew to 80–90% confluence. And then oil-red O staining working solution was added to cells. Nuclei were stained with hematoxylin and washed with PBS for 3 times. The cells were covered with PBS and photographed under microscope.

**Mitochondrial DNA copy number**

GeneJet Genomic DNA purification kit (Thermo Scientific, MA, USA) was used to purify total DNA from mice livers according to the manufacturer’s instruction. Mitochondrial DNA copy number was detected with mitochondrial DNA copy number assay kit (Detroit R&D, MI, USA) by qPCR as manufacturer's instruction.

**ATP level detection**

Cellular ATP levels were measured using a firefly luciferase-based Bioluminescence ATP assay kit (Beyotime, Jiangsu, China). Briefly, cells were lysed and centrifuged at 12,000g for 5 min at 4 C. Then 100 μL of each supernatant was mixed with 100 μL of ATP detection solution. Luminance (RLU) was measured by a Luminometer (Promega, Madison, WI).

**Hepatic triglyceride (TG) content detection**

Liver tissues of mice were homogenized, the protein concentration of each sample was detected. And then the hepatic TG content was detected with a microplate reader colorimetric method using a TG assay kit (Nanjing Jiancheng Bioengineering Institute, Nanjing, China), according to the instructions of the reagent company.

**Mitochondrial OCR detection**

Mitochondrial OCR detection of hepatocytes were measured using the XF-24 analyzer (Seahorse Bioscience, US) according to the manufacturer’s instructions. Briefly, TRPM8-siRNA- or TRPM8-plasmid (overexpression, OE)-transfected L02 cells were seeded into XF-24 microplates at 37°C with 5% CO2. Cells were maintained at 37°C in a non-CO2 incubator for 1 h, and then the basal OCR of the cells were measured. The OCR was normalized to the protein content.

**Primary hepatocyte culture and cell viability assay.**

Primary hepatocytes were prepared from male TRPM8 knockout (KO) mice and Wide type C57BL/6 (WT) mice using the collagenase perfusion method as previously described(1). Cell viability was measured by MTT assay.

**Statistical analysis**

Statistical significance was evaluated using one-way (for single variant) or two-way (for multiple variants) ANOVA analysis of variance followed by Bonferroni adjustment for multiple comparisons. Two groups were compared by the two-tailed Student’s unpaired t-test. Data are expressed as mean ± SEM. Differences were considered significant at a P value<0.05. All statistical tests justified as appropriate, and the data meet the assumptions of the tests. The variance is similar between the groups that are being statistically compared.

1. Liu G, Xie C, Fang Y, Qian K, Liu Q, Liu G, et al. Splenectomy after partial hepatectomy accelerates liver regeneration in mice by promoting tight junction formation via polarity protein Par 3-aPKC. Life Sci. 2018;192:91-8.

**Figure legends**

**Fig. S1.** **Liver function and the inflammatory response after PH are attenuated in TRPM8-deficient mice.**

(A) Representative H&E staining of liver Sections 24 hours after PH. (B) Elevation of AST and ALT levels in TRPM8 KO mice after PH. (C) The mRNA levels of TNF-α and IL-6 in the liver lysates of TRPM8 KO and WT mice 24 hours after PH. Data are presented as the mean ± SEM (n=5). *p<0.05, **p<0.01, vs. WT after PH at the indicated time.

**Fig. S2. TRPM8 contributes to the proliferation and cell cycle of hepatocytes in vitro**

(A-B) The expression level of TRPM8 after knockdown was identified by PCR and Western blotting. Data are expressed as the mean ± SEM, n = 3, **p < 0.01, ***p < 0.001 compared to siControl. (C) The proliferation of TRPM8-siRNA- or TRPM8-plasmid (OE)-transfected L02 cells was determined by EdU assays. The data are expressed as the mean ± SEM, n=5. **p* < 0.05, ***p* < 0.01. (D) The cell cycle distribution was measured by flow cytometry. The data are expressed as the mean ± SEM, n=5. **p* < 0.05. (E) Representative images of Mito-Sox staining in TRPM8-siRNA- or TRPM8-plasmid (OE)-transfected L02 cells. The data are expressed as the mean ± SEM, n=5. **p* < 0.05, ****p* < 0.001. (F) Representative images of Oil Red O staining.

**Fig.S3. TRPM8 contributes to the proliferation of hepatocytes via mitochondrial metabolism mediated by PGC1α**

(A) The expression level of PGC1α of TRPM8-siRNA- or TRPM8-plasmid (OE)-transfected L02 cells was determined by Western blotting. (B) Intracellular Ca^2+^ levels were assessed in cells with Fluo-4 AM using a fluorescence microscope. (C) The protein levels of NRF1, TFAM, Aco2 and ATP5A in the liver lysates of mice 24 hours after PH. (D) The copy number of mitochondrial DNA was checked by PCR in the liver lysates of mice 24 hours after PH. The data are expressed as the mean ± SEM, n=5. ***p* < 0.01.

**Fig. S4.** **Effect of a TRPM8 agonist on liver function and the inflammatory response after PH**

(A) Elevation of AST and ALT levels in TRPM8 agonist-treated mice after PH. (B) The mRNA levels of TNF-α and IL-6 in the liver lysates of TRPM8 agonist-treated mice 24 hours after PH. Data are presented as the mean ± SEM (n=5). **p*<0.05, ***p*<0.01, vs. WT after PH at the indicated time.

**Fig. S5. Pharmacological effects of a PGC1α agonist (Zln005) on liver regeneration.**

(A) Plasma ALT and AST levels in mice at the indicated times after PH were detected. (B) The mRNA levels of TNF-α, IL-6 and IL-1β in the liver lysates of mice 24 hours after PH. Data are presented as the mean ± SEM (n=5). **p*<0.05, ***p*<0.01 vs. WT after PH at the indicated time.
