# Supplementary figures and images for: TRPM8 contributes to liver regeneration via mitochondrial energy metabolism mediated by PGC1α
Source: Cell Death Dis. 2022 Dec 16;13(12):1050. doi: 10.1038/s41419-022-05475-4 (PMC9758188; doi:10.1038/s41419-022-05475-4)

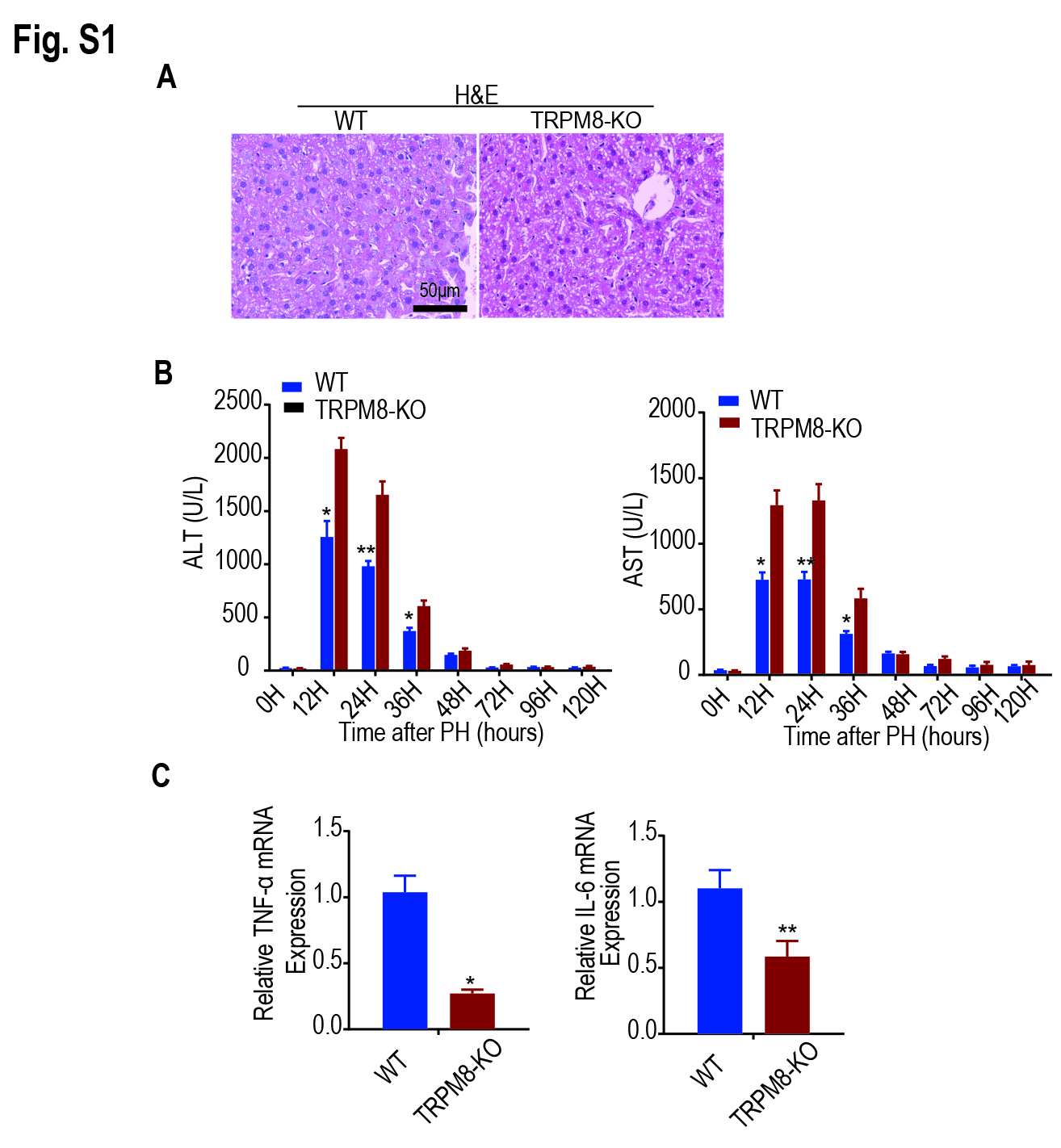

Supplement: Supplementary file 2 — Supplementary Figure S1 [file 41419_2022_5475_MOESM2_ESM.tif]

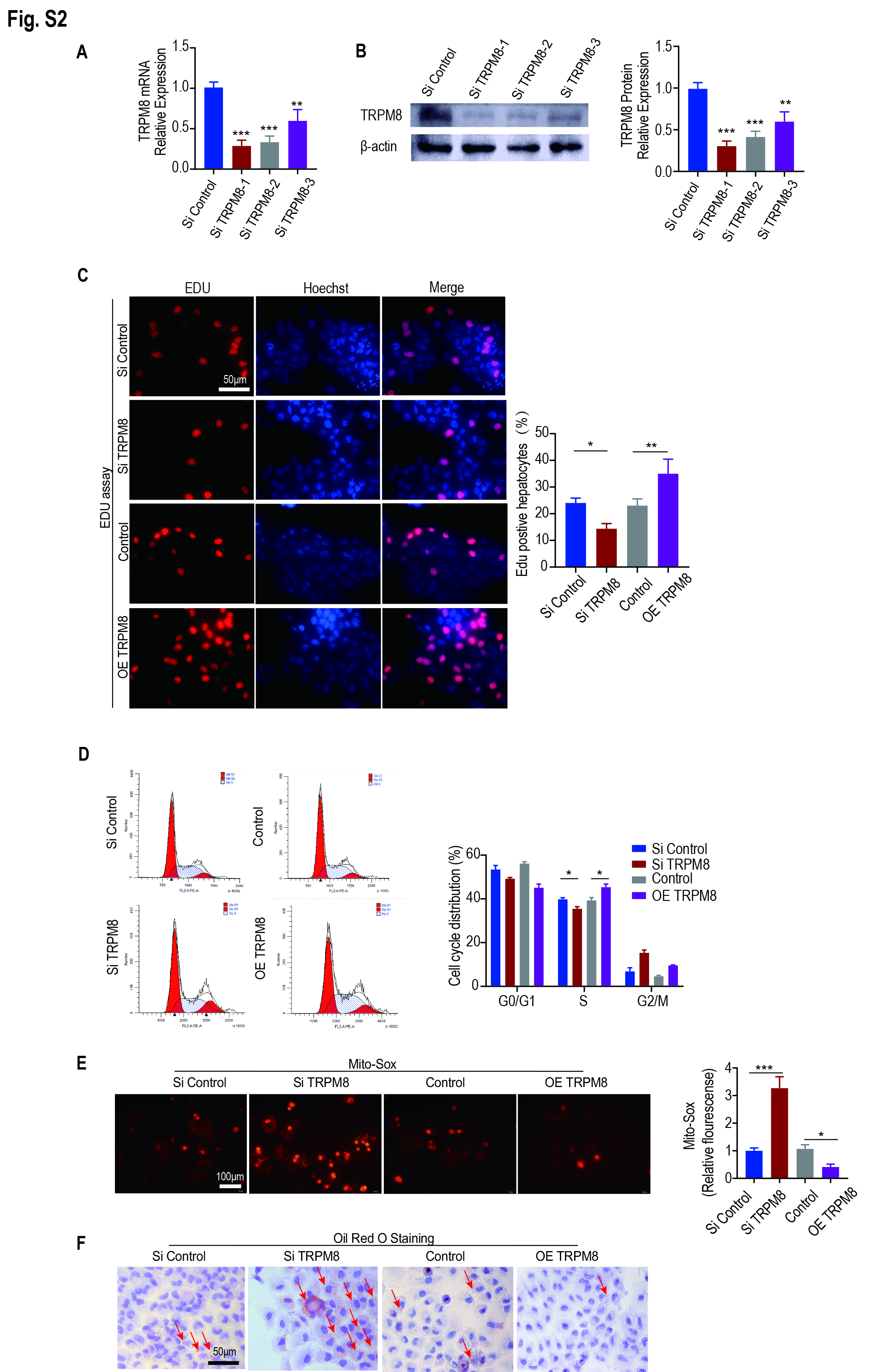

Supplement: Supplementary file 3 — Supplementary Figure S2 [file 41419_2022_5475_MOESM3_ESM.tif]

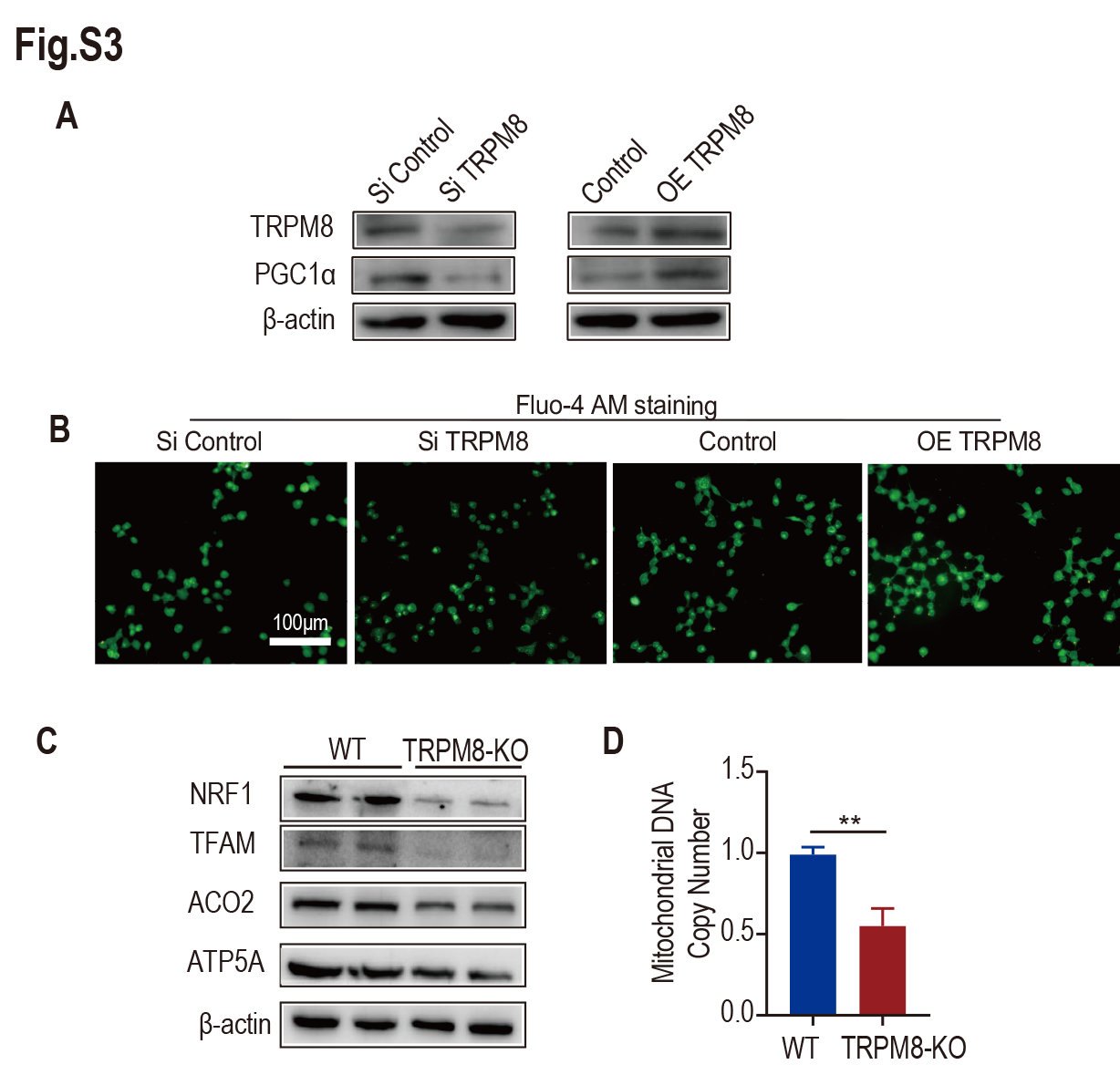

Supplement: Supplementary file 4 — Supplementary Figure S3 [file 41419_2022_5475_MOESM4_ESM.tif]

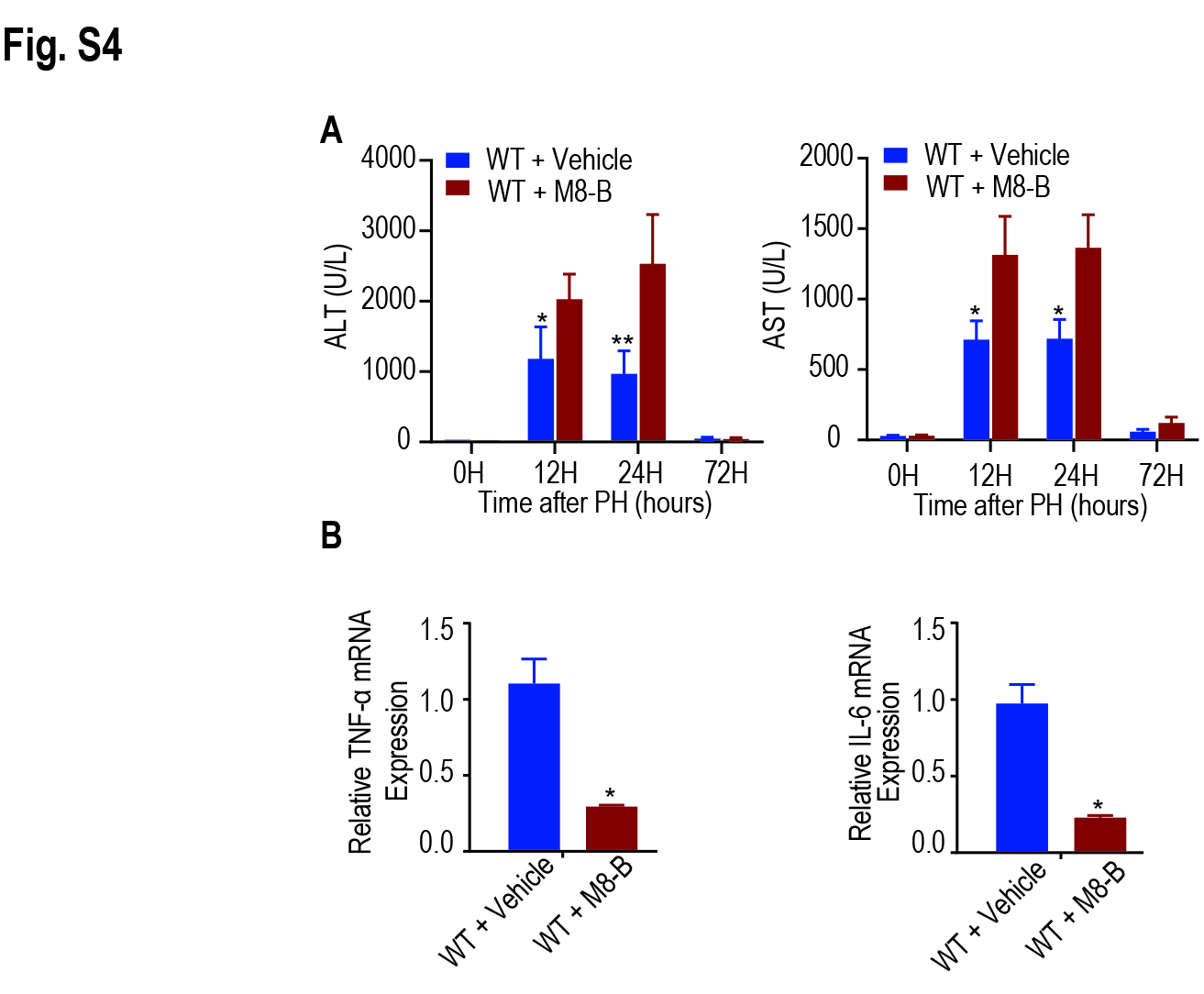

Supplement: Supplementary file 5 — Supplementary Figure S4 [file 41419_2022_5475_MOESM5_ESM.tif]

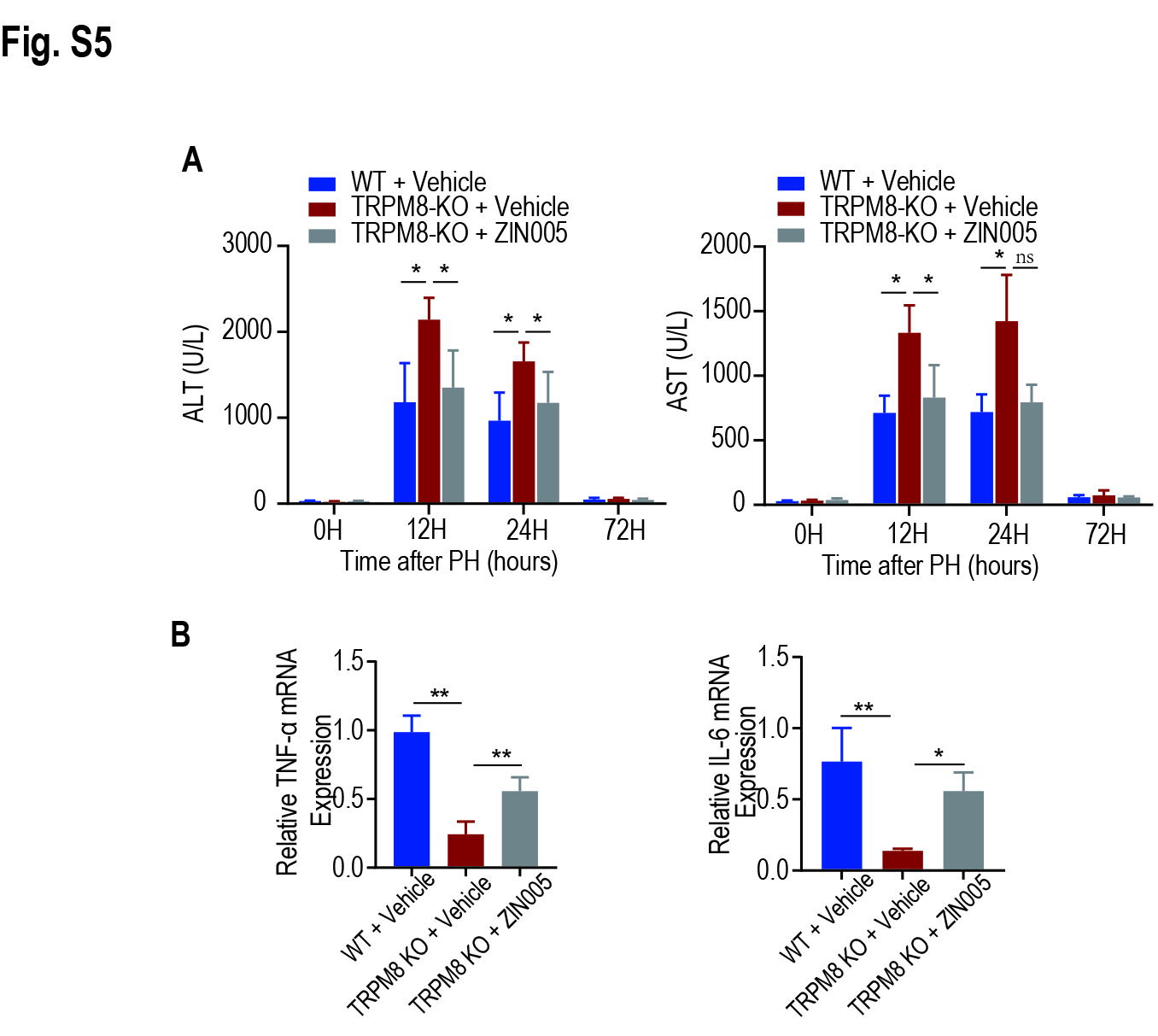

Supplement: Supplementary file 6 — Supplementary Figure S5 [file 41419_2022_5475_MOESM6_ESM.tif]

Fig. 3

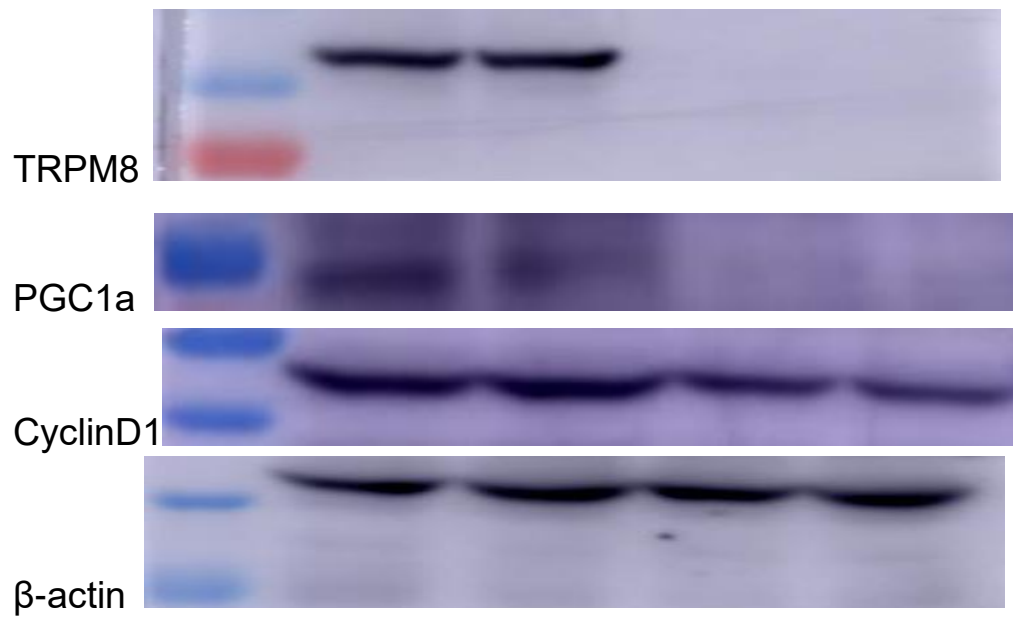

Fig. 4

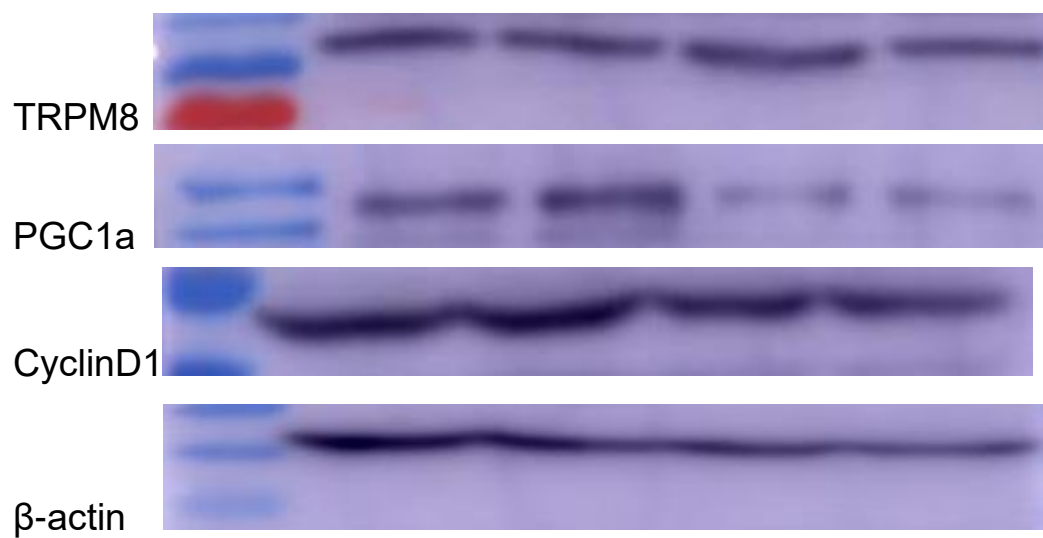

Fig. 5

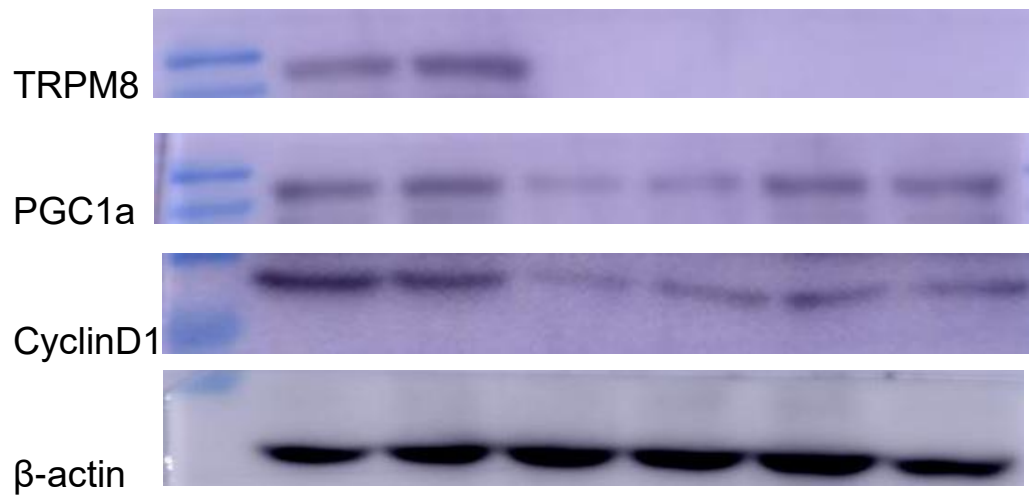

Fig. S2

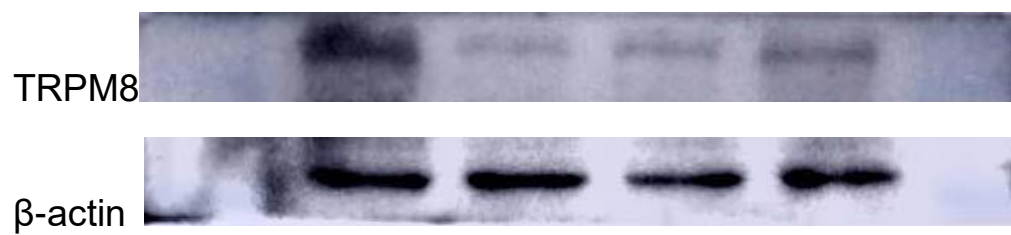

Fig. S3A

Left

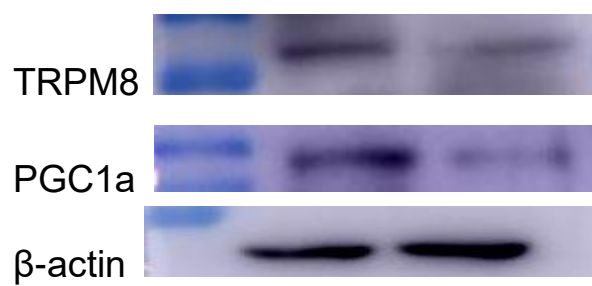

Right

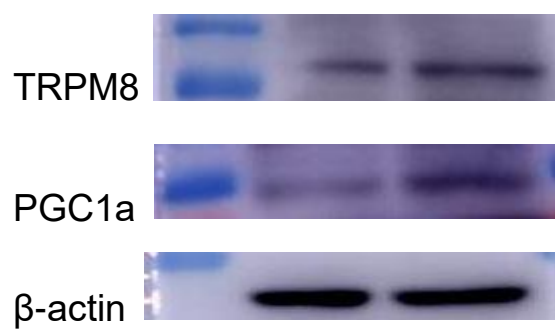

Fig. S3C

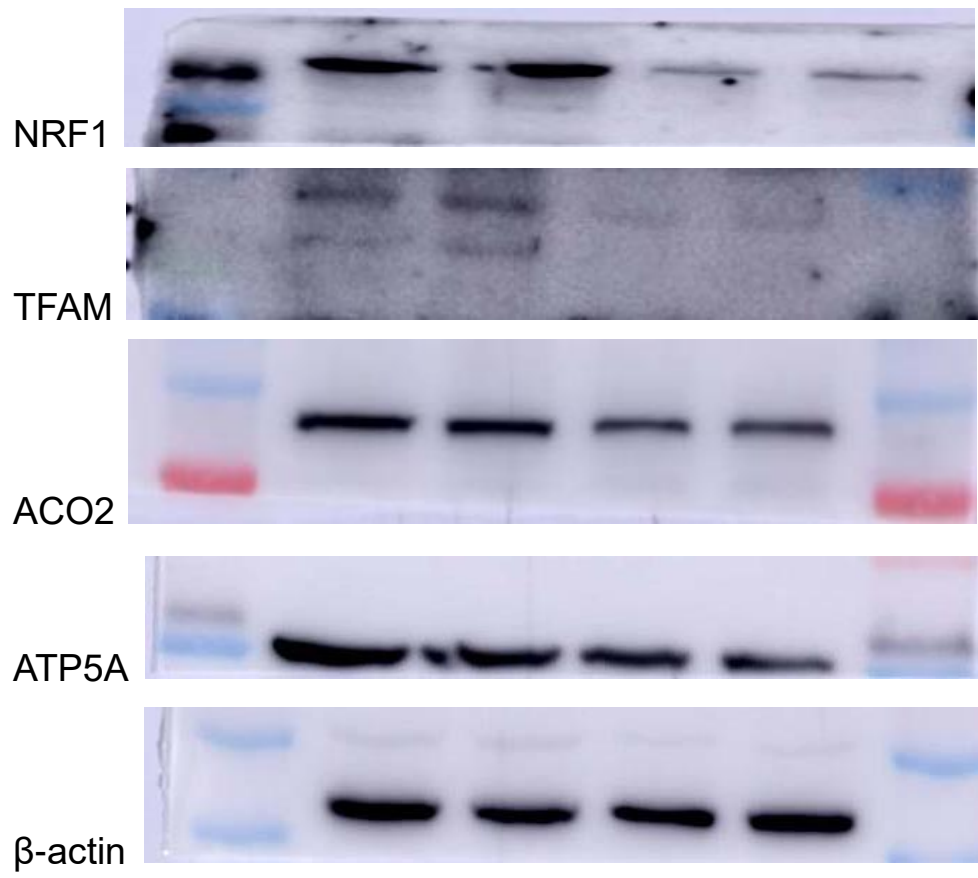

Supplement: Supplementary file 7 — Original Data File [file 41419_2022_5475_MOESM7_ESM.pdf]
